# Assessment of genetic variation for pathogen-specific mastitis resistance in Valle del Belice dairy sheep

**DOI:** 10.1186/s12917-016-0781-x

**Published:** 2016-07-28

**Authors:** Marco Tolone, Cristian Larrondo, José M. Yáñez, Scott Newman, Maria Teresa Sardina, Baldassare Portolano

**Affiliations:** 1 Dipartimento Scienze Agrarie e Forestali, Università degli Studi di Palermo, Viale delle Scienze, Palermo, 90128 Italy; 2 Faculty of Veterinary and Animal Sciences, University of Chile, Av. Santa Rosa, La Pintana, Santiago 11735 Chile; 3 Genus plc, Hendersonville, TN 37075 USA

**Keywords:** Ewes, Mastitis, Pathogen, Resistance

## Abstract

**Background:**

Mastitis resistance is a complex and multifactorial trait, and its expression depends on both genetic and environmental factors, including infection pressure. The objective of this research was to determine the genetic basis of mastitis resistance to specific pathogens using a repeatability threshold probit animal model.

**Results:**

The most prevalent isolated pathogens were coagulase-negative staphylococci (CNS); 39 % of records and 77 % of the animals infected at least one time in the whole period of study. There was significant genetic variation only for *Streptococci* (STR). In addition, there was a positive genetic correlation between STR and all pathogens together (ALL) (0.36 ± 0.22), and CNS and ALL (0.92 ± 0.04).

**Conclusion:**

The results of our study support the presence of significant genetic variation for mastitis caused by *Streptococci* and suggest the importance of discriminating between different pathogens causing mastitis due to the fact that they most likely influence different genetic traits. Low heritabilities for pathogen specific-mastitis resistance may be considered when including bacteriological status as a measure of mastitis presence to implement breeding strategies for improving udder health in dairy ewes.

## Background

Mastitis is one of the most common diseases affecting dairy sheep. Mastitis leads to major economic losses, mainly due to discarded milk, reduced milk production and quality, alteration of cheese-making properties, early culling, and increased health care costs [1–8]. The alterations or reductions of the dry matter of milk and its composition, have a substantial effect on the economic and industrial values of the milk, considering that almost all is processed into fermented products and cheeses [4, 9, 10]. Mastitis resistance is a complex and multifactorial trait, and its expression depends on both genetic and environmental factors, including infection pressure. In the broadest sense, resistance could be defined as the ability to avoid any infection and/or the quick recovery from an infection [11, 12], and involves different factors such as to avoid entry of the pathogen into the mammary gland, to induce an immune response capable of limiting pathogen development in the udder and to recover from the infection, as well as controlling the pathogenic effects of the infection, such as tissue damage [13].

Over 100 different micro-organisms can cause mastitis, in particular coliform bacteria, staphylococci and streptococci [14]. In dairy sheep the most important agents involved in clinical mastitis are the bacterial infections, and the most frequently isolated pathogens are coagulase-negative staphylococci (CNS); that are present on and around the udder skin [9] with a different pathogenicity causing clinical and subclinical mastitis [15–18]. The bacterial pathogens responsible for infection of the mammary gland may be grouped into two main categories: major and minor pathogens. Major pathogen infection generally results in clinical illness or strong inflammatory responses and reduced milk yields, whereas minor pathogen infection is usually subclinical [19].

Selection for genetic resistance to mastitis can be done directly or indirectly. Direct selection corresponds to the diagnosis of the infection: the actual trait [i.e., bacteriological examination of milk and/or observation of clinical cases of mastitis] is measured on the animal or its relatives. Indirect selection corresponds to a prediction of the bacteriological status of the udder based on traits related to the infection [e.g., inflammatory parameters]: an indicator trait for mastitis is measured on the animal itself or its relatives [20]. Simple and indirect methods have been widely applied based on the evaluation of the degree of inflammation or of internal mammary lesions [21]. Their accuracy was established by bacteriological analysis as a reference method [17]. Among the indirect methods, the most frequently used to detect mastitis are milk somatic cell count (SCC). SCC is considered as a good measure to indirectly select for mastitis resistance in cattle, especially when a direct measure of clinical mastitis incidence is not available [18, 22]. In cattle, values of SCC between 250 and 300 × 10^3^ cells/mL are recommended as satisfactory discrimination thresholds to distinguish between healthy and infected udders. In sheep there is no widely accepted threshold [15, 23] but some studies suggested a critical limit of 500 × 10^3^ cells/mL [24]. There are few studies concerning genetic variation of mastitis in sheep according to bacteriological status [22, 25]. A genetic selection approach could be one of the strategies for controlling mastitis and has been shown to be a valid option, together with management, to prevent mastitis cases [1, 26]. Studies have reported genetic variation accounting for resistance to mastitis in Valle del Belice dairy sheep [22, 24]. These authors have defined mastitis as a binary trait distinguishing between ewes with at least one case of mastitis (1) and ewes without (0) in a defined period of lactation and was analyzed using a linear model approach. This definition excluded alternative definitions, for example multiple cases of mastitis within lactation, and ignored the etiology of intra-mammary infections. The purpose of this study was to determine the genetic bases of pathogen-specific resistance to mastitis in Valle del Belice dairy sheep using a threshold repeated model.

## Methods

### Data

Data were collected between 2006 and 2011 in five Valle del Belice flocks, with a total of 2350 ewes and 5856 animals in the pedigree. Observations for this study included 1795 primiparous, 1285 secondiparous and 2225 multiparous dairy ewes. All ewes were milked twice daily (morning and evening), and records for milk yield (MY), bacteriological status (infected or not infected), and SCC were collected at approximately monthly intervals, following an A4 recording scheme (monthly records with two daily milking) which is defined by the International Committee for Animal Recording [27]. The milk samples were collected during routine milking so avoiding any harmful process to individuals. The consent for sample collection was obtained by the animals’ owners. Moreover, Sample collection, animal management and cares were in agreement with the Directive 2010/63/EU. The observed bacteriological colonies were identified as: *Escherichia coli* (ESCCL), *Staphylococcus aureus* (STHAU), *Streptococcus dysgalactiae* (STPDG), *Streptococcus uberis* (STPUB), *Streptococcus agalactiae* (STPAG) and *Bacillus spp.* (BACIL), *Corynebacterium spp.* (CORLT), *Pasteurella spp.* (PASCL), *Pseudomonas spp.* (PSELT), coagulase negative staphylococci (CNS) and *Streptococcus spp.* (STR). Ewes were considered infected if at least one record with positive bacteriological test during lactation period was recorded, while they were considered healthy if the bacteriological test did not show a positive result. Ewes were measured more than one time during the same lactation. Thus, the repeatability of records is across and within lactations. Table 1 shows average number of records per ewe within lactations. Moreover, ewes were considered infected if more than five colony forming units (CFU) per 10 μl of milk of one species of bacteria were isolated. The response variable used in the model corresponds to the binary disease status, coded as 0 or 1 to represent uninfected or infected individuals, respectively.

**Table 1:** Mean, standard deviation (SD), minimum (Min) and maximum (Max) number of records per ewe within lactation

Lactation	Mean	SD	Min	Max
1	3.47	1.97	1	12
2	4.13	2.25	1	20
3	4.46	2.33	1	15
4	4.75	2.4	1	12
5	7.47	5.75	1	32

### Trait definition and statistical model

Phenotypic observations of infection status were defined as a repeated binary trait. The binary trait distinguished between sheep with infected udder status (1) and uninfected udder status (0) within each lactation for each particular pathogen described above. SCC was also recorded and normalized through a logarithmic transformation into somatic cell score (SCS) according to the formula of Ali and Shook [28]:

$$ \mathrm{Somatic}\ \mathrm{cell}\ \mathrm{score}\ \left(\mathrm{S}\mathrm{C}\mathrm{S}\right) = { \log}_2\left(\mathrm{S}\mathrm{C}\mathrm{C}/100,000\right) + 3 $$

The binary trait was analyzed using the following repeatability threshold probit animal model:

$$ \boldsymbol{Pr}\left({\boldsymbol{Y}}_{\boldsymbol{i}\boldsymbol{jklmn}}\right) = \boldsymbol{\varPhi} \left(\boldsymbol{\mu} +\boldsymbol{O}{\boldsymbol{P}}_{\boldsymbol{i}}+\boldsymbol{M}{\boldsymbol{Y}}_{\boldsymbol{j}} + \boldsymbol{F}\boldsymbol{Y}{\boldsymbol{S}}_{\boldsymbol{l}}+\boldsymbol{P}{\boldsymbol{E}}_{\boldsymbol{m}}+{\boldsymbol{A}}_{\boldsymbol{n}}\right) $$

Where *y*_*ijklmn*_ is the observation for the specific pathogen causing mastitis (CNS, STR, ESCCL, STHAU, STPDG, STPUB, STPAG, BACIL, CORLT, PASCL, PSELT and ALL); Φ is the normal cumulative density function; *μ* is the fixed effect of the overall mean; *OP*_*i*_ is the order of parity fitted as fixed effect (with 5 classes); *MY*_*j*_ is the milk production yield fitted as covariate; *FYS*_*l*_ is the flock-year-season random effect (51 classes); *PE*_*m*_ is the random permanent environmental effect of the individual *m* across lactations (2350 levels with records); and *A*_*n*_ is the random animal effect (5856 levels in the pedigree). The implicit residual variance on the underlying scale is 1 for the probit model (standard normal). Parameters of the univariate threshold models were estimated using ASREML version 3.0 [29].

### Heritabilities

Heritabilities for resistance to different pathogens were calculated as:

$$ {\boldsymbol{h}}^2=\frac{\sigma_a^2}{\sigma_a^2+{\sigma}_{FYS}^2+{\sigma}_{PE}^2 + {\sigma}_e^2} $$

Where *σ*_*a*_^2^ is the animal additive genetic variance, *σ*_*FYS*_^2^ is the variance associated with flock-year-season, *σ*_*PE*_^2^ is the variance due to permanent environment and *σ*_*e*_^2^ is residual variance. For all traits, the animal effect was assumed ~ **N**(0, **A***σ*_*a*_^2^), where **A** is the additive genetic relationship matrix among all animals included in the pedigree (5856). Similarly, FYS and PE effects were assumed ~ **N**(0, **I***σ*_*FYS*_^2^) and ~ **N**(0, **I***σ*_*PE*_^2^) , where **I** is an identity matrix with order equal to number of FYS and PE classes (51 and 2350) respectively.

## Results

Arithmetic mean and standard deviation of MY, SCC and SCS, for infection status (infected and uninfected) of udders are shown in Table 2. Daily average MY values were 1275 ± 544 and 1338 ± 558 g, for infected and uninfected udders, respectively. Mean SCC was 2908 ± 4926 and 1155 ± 3083 (x 10^3^ cells/mL), for infected and uninfected udders, respectively; whereas mean SCS was 6.21 ± 2.45 and 4.55 ± 2.13. Overall mean values for both infected and uninfected udders were 1314 ± 553 g, 1815 ± 3972 (x 10^3^ cells/mL), and 5.18 ± 2.3, for MY, SCC and SCS, respectively.

**Table 2:** Arithmetic mean and SD for MY, SCC, and SCS of infected and uninfected udders

	n	MY(g)	SCC(x 10^3^) ^a^	SCS
		Mean ± SD	Mean ± SD	Mean ± SD
Infected	8019	1275 ± 544	2908 ± 4926	6.21 ± 2.45
Not infected	13,246	1338 ± 558	1155 ± 3083	4.55 ± 2.13
All	21,265	1314 ± 553	1815 ± 3972	5.18 ± 2.33

*SD* standard deviation, *MY* milk yield, *SCC* somatic cell count, *SCS* somatic cell score

*n* number of observations

^a^ Cells/mL of milk

There was a low prevalence of isolation due to CORLT, ESCCL, PASCL, PSELT, STPDG, STPUB, STPAG, and BACIL, in all the observations and according to infection status of animals. Models did not converge for these pathogens, most likely due to the low incidence (zero inflation).

Absolute (AF) and relative (RF) frequency distribution according to infection status (infected or uninfected) observations within all the records data set, are shown in Table 3. The most prevalent isolated pathogens were CNS with 7951 (39 %) observations, followed by STHAU (940; 5 %) and STR (541; 3 %). Considering all pathogens (ALL), 8019 (38 %) milk samples were infected.

**Table 3:** Absolute (AF) and relative (RF) frequency distribution according to udder status of observations (*n* = 20,519)

Pathogen	Status ^a^	AF	RF
CNS	0	12,568	0.61
	1	7951	0.39
STHAU	0	19,579	0.95
	1	940	0.05
STR	0	19,978	0.97
	1	541	0.03
ALL	0	13,246	0.62
	1	8019	0.38

*CNS* coagulase-negative *staphylococci*, *STHAU Staphylococcus aureus*, *STR streptococci*, *ALL* observed bacteriological colonies as described in M&M section

^a^ Udder status as binary record: 0 = not infected, 1 = infected

Table 4 shows absolute (AF) and relative (RF) frequency distributions according to udder status (infected or uninfected) of animals per pathogen. Similarly, the most prevalent isolated pathogens affecting ewes were CNS (1811; 77 %), followed by STHAU (513; 21.83 %) and STR (217; 9.23 %) when only animals were analyzed.

**Table 4:** Absolute (AF) and relative (RF) frequency distribution according to udder status of animals (*n* = 2350) per pathogen

Pathogen	Status ^a^	AF	RF
CNS	0	539	0.23
	1	1811	0.77
STHAU	0	1837	0.78
	1	513	0.22
STR	0	2133	0.91
	1	217	0.09
ALL	0	612	0.26
	1	1738	0.74

*CNS* coagulase-negative *staphylococci*, *STHAU Staphylococcus aureus*, *STR streptococci*, *ALL* observed bacteriological colonies as described in M&M section

^a^ Udder status as binary record: 0 = not infected, 1 = infected

Table 5 shows components of variance due to Flock-Year-Season effect, permanent environment effect, additive genetic effect, phenotypic effect and heritabilities for resistance to CNS, STR and ALL. Due to the low frequency of isolation of ESCCL, STHAU, STPDG, STPUB, STPAG, BACIL, CORLT, PASCL, and PSELT in the total records and isolation of pathogens per animals (Tables 3 and 4), problems associated with convergence were found when analyzing resistance to these bacteria. In contrast, there was statistically significant genetic variation for CNS, STR, and ALL pathogens (Table 5). Variances due to FYS random effect were 0.18, 0.09 and 0.79, whereas due to permanent environment effect were 0.39, 0.39, and 0.40 for CNS, STR and ALL, respectively. Variances of additive and phenotypic effects were 0.03, 0.15, 0.04; and 1.60, 1.62, 2.23, for CNS, STR and ALL, respectively. The heritability obtained for STR (0.09) was significant different from zero, whereas for CNS (0.02) and ALL (0.02) were not.

**Table 5:** Estimates of components of variance and their standard errors for infectious status

Resistance Trait	**σ** ^2^_**FYS**_	**σ** ^2^_**PE**_	**σ**_**a**_^2^	**σ**_***P***_^2^	*h* ^2^
Infection Status					
ALL	0.79 ± 0.17	0.40 ± 0.03	0.04 ± 0.02	2.23 ± 0.17	0.02 ± 0.01
CNS	0.18 ± 0.04	0.39 ± 0.03	0.03 ± 0.02	1.60 ± 0.05	0.02 ± 0.01
STR	0.09 ± 0.03	0.39 ± 0.07	0.15 ± 0.07	1.62 ± 0.05	0.09 ± 0.04

*σ*_*FYS*_^2^ Flock-Year-Season effect, *σ*_*PE*_^2^ permanent environment effect, *σ*_*a*_^2^) additive genetic effect, *σ*_*P*_^2^ phenotypic effect, *h* ^2^ heritabilities

*CNS* coagulase-negative *staphylococci*, *STR streptococci*, *ALL* observed bacteriological colonies as described in M&M section

Table 6 shows phenotypic and genetic correlations for resistance to mastitis caused by STR, CNS and ALL estimated using a multivariate repeatability linear model. All of the estimated phenotypic and genetic correlations were significantly different from zero. The phenotypic correlation between ALL and STR was low, however, the genetic correlation between these traits was moderately high indicating that there was a direct relationship between these traits in genetic terms. On the other hand, both phenotypic and genetic correlations between ALL and CNS were high, indicating that there was a strong positive relationship, both phenotypic and genetic, between these traits. These results suggest that resistance to CNS is a similar to the resistance when it is measured as ALL. In addition, the phenotypic correlation between STR and CNS was negative and the genetic correlation between these traits was low, thus, indicating that selection for improved STR will not have an impact on CNS resistance.

**Table 6:** Phenotypic (above diagonal) and genetic (below diagonal) correlations and standard errors for resistance to mastitis

Infection status	STR	CNS	ALL
STR	-	−0.08 ± 0.02	0.17 ± 0.01
CNS	0.24 ± 0.25	-	0.87 ± 0.01
ALL	0.36 ± 0.22	0.92 ± 0.04	-

*CNS* coagulase-negative *staphylococci*, *STR streptococci*, *ALL* observed bacteriological colonies as described in M&M section

## Discussion

The overall infection prevalence considering frequency of infection on all the samples of records was 37.7 %, close to the value of 42 % reported in a previous investigation in the same breed [25], and higher than the values of 26.2 and 24.6 % reported by Pengov [15] and Gonzalo et al. [16], respectively. However, in the study of Pengov [15] only one milk sample (*n* = 496 samples, 251 ewes) of udder halves was considered, whereas the study of Gonzalo et al. [16] was based only on subclinical mastitis prevalence. When the prevalence was assessed on all the animals it increased to 74 %, higher than any prevalence reported in previous studies.

Probably the high SCC reported in our study are a consequence of inadequate preventive management, a lack of strict hygiene conditions and extensive management practices, generating a high number of subclinical mastitis cases due to environmental pathogens. Moreover, our results suggested that ewes have higher SCC than cows and it is therefore necessary to establish an acceptable threshold in dairy sheep considering the difference in SCC between breeds and other factors [15, 18, 22].

Leitner et al. [9] suggested categories for classification of SCC in sheep and goat related to quality of milk and infection status. These researchers suggested that infection of 25, 50 and 75 % of the udders in a given herd was associated with 4.1 to 12.2 % of milk loss in sheep and 0.8 to 2.3 % in goats [9]. Mavrogenis et al. [30] suggested that an increase of 0.5 cells/mL × 10^6^ SCC above the mean resulted in reduction of mean individual daily production of milk by 18 g.

In the present study, there was a 68 g difference in mean MY between infected and non- infected ewes. For SCC, the mean value for infected animals was approximately 3-fold higher than uninfected animals, similar to values reported in a previous study [24]. However mean SCC for healthy animals were different. Mean SCC for uninfected animals was different to the value of 89 cells/mL × 10^3^ reported by Pengov [15] and 311 cells/mL × 10^3^ reported by Leitner et al. [4], and similar to the value of 1490 cells/mL × 10^3^ reported by Kern et al. [31]. These studies focused on Domestic Highland, East Friesand, and Awassi breeds, including their crosses and the Assaf breed, respectively. Moreover, considering the whole data set, mean SCC for infected animals was similar to reported values in the literature [4, 24]. Mean SCS for uninfected and mean SCS of whole data set were similar to the values reported in Valle del Belice [24, 25], and Churra dairy sheep breeds [32]. For mean SCS of infected animals, Riggio et al. [24] reported a value of 6.42 and Leitner et al. [33] a value of 6.32 in Israel-Assaf and Awassi sheep, similar to the value of 6.21 obtained in our research. Another study reported lower values of mean SCS of infected animals using different breeds [32].

Our study confirms that CNS is the most prevalent etiological group of bacteria in the infected dairy ewes. The frequency of isolation of CNS on record (39 %) was lower than other studies ranging from 60 to 90 % [3, 24, 32, 34–36]. Moreover, a high percentage (77 %) of animals were found infected at least one or more times in the period of study, showing the importance of this group of bacteria in this population.

Most cases of CNS infection produce subclinical mastitis, although intramammary infections in its subclinical form by CNS have been described as the main single factor affecting udder health and profitability in small ruminants [9]. Besides, due to the high prevalence of CNS during the ewe’s lactations, subclinical cases could persist, significantly increase SCC and consequently cause clinical mastitis. This is a possible explanation of the observed differences of SCC between infected and non-infected animals and frequency of animals infected by CNS. Moreover, considering the opportunistic nature of CNS [17], with adequate hygiene practices, correct milking routine and periodic revision of milking equipment, intramammary infections by CNS could be reduced.

In this investigation, CNS were classified as minor pathogens. Researchers have reported that some species of CNS can cause high SCC, similar to those of major pathogens [15, 32, 37] and even clinical mastitis [32, 38]. Ariznabarreta et al. [32] described that *Staphylococcus caprae* and *S. simulans* were associated with high log SCC, 6.43 and 6.35, respectively, in contrast with other CNS bacteria such as *S. chromogenes*, *S. hominis*, *S. capitis*, *S. haemolyticus* and *S. epidermidis* ranging from 5.93 to 6.09. Therefore, there was variation in the inflammatory response according to the involved CNS species and their pathogenicity in milk measured through SCC, even on average ten times higher than in dairy cattle [15]. STHAU was the second one more frequently isolated bacteria in our study (5 %) followed by STR (3 %). This order differs from other authors, which reported that CNS is the most prevalent group of bacteria followed by STR [15, 31, 35, 38]. For STHAU, ewes infected at least one time or more in the period of study were 22 % (513). These findings are different respect to what reported by Riggio et al. [24] with values of 10.47 % of milk samples and similar to other studies ranging from 2 to 5.5 % [15, 32]. Infection due to STHAU is related with subclinical to acute clinical mastitis (gangrenous mastitis) with different clinical symptoms according to the virulence of the strains and in severe cases lead towards culling of the affected sheep [2, 17]. The high percentage of animals affected by STHAU in the period of study could be related with clinical mastitis cases and culling of ewes in this population. This was in agreement with Mavrogenis et al. [30] which identified STHAU as the most prevalent bacteria in clinical mastitis cases.

In sheep a heritability estimate of 0.09 for infection status assessed by bacteriological analyses was reported by Riggio et al. [24] and Tolone et al. [25] in the Valle del Belice breed using a threshold animal model assuming a probit link function. Gonzalo et al. [39] estimated genetic parameters of SCC in Churra sheep considering the type of mammary pathogen using a multitrait repeatability animal model. They reported that the effect related to the type of pathogen accounted for 32.5 % of the total variance in SCC, a value similar to that obtained for the residual effect (34.9 %), indicating a high relative importance of the type of pathogen in the decomposition of the variance for SCC. In addition, Holmberg et al. [40] in dairy cattle reported genetic variances for different pathogens ranging from 0.024 to 0.188, similar to the values of the present research (0.03 to 0.15). These results showed the importance of differentiating between the types of mammary bacteria assessed by bacteriological analyses in genetic mastitis studies.

Variances due to permanent environment and FYS effects were high and were important factors to explain the phenotypic variance resistance against CNS, STR and ALL. The possible explanations of these results for CNS group of pathogens are their nature and their high frequency of isolation in this sheep breed. CNS group of bacteria are related with inadequate management and hygiene practices, which could be different among the flocks, through the year and among them. Therefore, due to opportunistic nature of CNS, poor flock management and inadequate milking hygiene could increase the probability of occurrence of mastitis, and flocks may act as reservoirs of some CNS species. Taking into account the predominant sheep husbandry system in Sicily based on grazing with animals kept outdoors, reductions in pasture quantity and quality through the year as in summer (peak of lambing in Valle del Belice sheep) could be a stress factor to increase the susceptibility due to ALL infection. High temperatures in summer are associated with heat stress [8], and as occurs in dairy cattle heat stress is recognized as a factor which increases susceptibility to mastitis. Gonzalo et al. [41] reported that month within flock and flocks were accounted 44.1 % of the variance on bulk tank bacteria count, whereas Portolano et al. [8] reported that flock-year of lambing effect explains 27 % of the variance of time interval between lambing and first record with mastitis.

Heritabilities for pathogen-specific mastitis were in agreement with results of De Haas [20] in dairy cattle ranging from 0.02 to 0.10. However, this study only included heritabilities of pathogens involved in clinical mastitis cases and were estimated through threshold and linear models. For genetic correlations, the one estimated between CNS and ALL (0.92) was positive and very high suggesting that both are the same traits. This could be explained for the high frequency of isolation of CNS in the records (77 %). Thus, a high percentage of ALL group is explained by CNS pathogens. Furthermore, due to the fact that phenotypic variation for CNS and ALL is determined primarily by an environmental component both type of traits (CNS and ALL) could be controlled more effectively by applying a correct management measures instead of selective breeding on these population.

In the Valle del Belice breed, where the current selection is mainly practiced on a “within farm” approach and based on own performance of ewes, it is unlikely that selection for mastitis resistance is successful, independent of the use of infection status or SCS.

## Conclusions

The results of our study support the presence of significant genetic variation for resistance to one specific pathogen causing mastitis (i.e. *Streptococci*). The high genetic correlation between ALL and CNS indicate that both are almost the same trait. The opportunistic nature of CNS and the high environmental influence of CNS resistance suggest that improvement of flock management and adequate milking hygiene could reduce significantly the incidence of mastitis caused by this pathogen in Valle del Belice dairy sheep.

## Abbreviations

A, animal; AF, absolute frequency; ALL, all pathogens together; BACIL, *Bacillus spp.*; CFU, five colony forming units; CNS, coagulase-negative staphylococci; CORLT, *Corynebacterium spp.*; ESCCL, *Escherichia coli*; FYS, flock year season; MY, milk yield; OP, order of parity; PASCL, *Pasteurella spp.*; PE, permanent environmental; PSELT, *Pseudomonas spp.*; RF, relative frequency; SCC, somatic cell count; SCS, somatic cell score; STHAU, *Staphylococcus aureus*; STPAG, *Streptococcus agalactiae*; STPDG, *Streptococcus dysgalactiae*; STPUB**,***Streptococcus uberis*; STR, *Streptococci*; STR, *Streptococcus spp*
